# Pituitary adenylate cyclase-activating polypeptide (PACAP)^+^ cells in the paraventricular nucleus of the thalamus: relationship with binge-type eating in male and female mice

**DOI:** 10.1007/s00213-024-06692-9

**Published:** 2024-09-28

**Authors:** Genevieve R. Curtis, Brody A. Carpenter, Breanne E. Pirino, Annie Hawks, George Li, Jessica R. Barson

**Affiliations:** Department of Neurobiology and Anatomy, Drexel University College of Medicine, 2900 Queen Lane, Philadelphia, PA 19129 USA

**Keywords:** Binge eating, C57BL/6 J, Cre, DREADDs, Ensure, mRNA, PCR, PVT, PACAP, Sex differences

## Abstract

**Rationale:**

Both the paraventricular nucleus of the thalamus (PVT) and the neuropeptide, pituitary adenylate cyclase-activating polypeptide (PACAP), are thought to be involved in food intake. Importantly, PACAP is expressed in cells of the PVT.

**Objectives:**

To determine if PACAP in cells of the PVT might mediate some of the involvement of the PVT with palatable food intake.

**Methods:**

In male and female C57BL/6 J mice and PACAP-Cre transgenic mice on a C57BL/6 J background, limited access to Milk Chocolate Ensure Plus® was used to establish a model of binge-type eating. Next, using quantitative real-time PCR, gene expression of PACAP in the PVT was measured in relation to this binge-type eating. Finally, using chemogenetics in PACAP-Cre transgenic mice, the effect of activation of PVT PACAP^+^ cells on binge-type eating was determined.

**Results:**

Males and females both engaged in binge-type eating with Ensure, although females engaged in this behavior to a greater degree than males. While females also had a higher baseline level of PVT PACAP mRNA than males, only males showed an increase in levels of PACAP after a history of exposure to Ensure, and only males showed a reduction in levels of PACAP immediately prior to a binge session. Conversely, activation of PACAP^+^ cells in the PVT reduced binge-type eating of Ensure, specifically in male mice.

**Conclusions:**

The present findings indicate that PVT PACAP^+^ cells influence and are influenced by binge-type eating. Thus, PACAP in the PVT might mediate some of the known involvement of the PVT with palatable food intake.

## Introduction

The paraventricular nucleus of the thalamus (PVT), a dorsal midline limbic nucleus, is thought to be involved in food intake (Barson et al. 2020; Iglesias and Flagel 2021; Kelley et al. 2005; McGinty and Otis 2020; Millan et al. 2017). In rats, activation of PVT cells and their projections has been found to increase sucrose seeking and intake (Barson et al. 2015, 2017; Labouebe et al. 2016) although, conversely, inactivation of PVT cells has also been found to increase sucrose seeking (Do-Monte et al. 2017) as well as chow intake (Stratford and Wirtshafter 2013). This discrepancy in effects may be due to the involvement of different PVT subregions or neurochemicals. One neuropeptide that is highly expressed in cells of the PVT is pituitary adenylate cyclase-activating polypeptide (PACAP) (Curtis et al. 2023, 2020; Gupta et al. 2018). Like the PVT itself, PACAP is known to be involved in food intake, as systemic injection of PACAP in mice has been found to inhibit intake of standard chow (Morley et al. 1992; Vu et al. 2015), and gene expression of PACAP in the hypothalamus is reduced by food deprivation and increased by chronic intake of a high energy diet (Hawke et al. 2009; Mounien et al. 2009). With preproPACAP independently processed into two peptide isoforms, PACAP-38 and PACAP-27 (Miyata et al. 1990), we have found that the less common isoform, PACAP-27, is the predominant isoform in cells of the PVT of both rats and mice, with expression of PACAP being greater in the posterior half of the PVT and in females than in males (Curtis et al. 2023; Gupta et al. 2018). Whether or not PACAP^+^ cells in the PVT are involved in food intake remains to be determined.

Binge eating disorder is the most common eating disorder in the United States (Udo and Grilo 2018). This disorder affects more women than men, but it is also the eating disorder with the most equal representation between women and men (Kessler et al. 2013; Udo and Grilo 2018). Clinically, binge eating disorder is characterized by the consumption of an unusually large amount of food in a short period of time, despite the absence of hunger and without compensatory behaviors (American Psychiatric Association 2022). The severity of this disorder is determined by the number of binge episodes per week (American Psychiatric Association 2022). Pre-clinically, two measures can be used to determine if eating can be classified as binge-type eating. Specifically, animals should (1) consume a larger than normal amount of food compared to a control group during the same period of time; and / or (2) escalate their intake across sessions (Babbs et al. 2012). While there is not a single, commonly-used model of binge-type eating in the field, many animal models induce binge-type eating by limiting access to a palatable food, typically one high in fat and / or carbohydrates (Bake et al. 2014; Berner et al. 2008; Boggiano et al. 2007; Corwin et al. 1998; Curtis et al. 2019; Dimitriou et al. 2000; Kreisler et al. 2017, 2018). These models do not always induce weight gain (Berner et al. 2008; Corwin et al. 1998; Curtis et al. 2019; Dimitriou et al. 2000; Kreisler et al. 2017). While animal models of binge-type eating have historically predominantly used rats, recent studies have begun to use mice (e.g. Bake et al. 2014; Hildebrandt et al. 2023; Scarpa and Bello 2023)). In both rats and mice, limited published work suggests that females binge at higher levels than males (Babbs et al. 2012; Freeman et al. 2021; Spierling et al. 2018).

The purpose of this study was to examine the role of PACAP^+^ cells in the PVT in food intake. Specifically, we sought to determine if PACAP in the PVT might mediate some of the known involvement of the PVT with palatable food intake. Using male and female mice, we first established our own model of binge-type eating, which could then be used in subsequent experiments. This model was developed to induce eating equivalent to moderate severity binge eating disorder (American Psychiatric Association 2022), by providing the palatable food in a cluster of days each week as has been observed in some clinical populations (Schreiber-Gregory et al. 2013). Next, we examined gene expression of PACAP in the PVT in relation to this binge-type eating. Finally, using chemogenetics in PACAP-Cre transgenic mice, we determined the effect of PACAP^+^ cell activation on binge-type eating. We hypothesized that females would engage in binge-type eating to a greater degree than males, that endogenous levels of PACAP mRNA would be reduced in the PVT prior to binge-type eating, and that enhanced activity in these PVT PACAP^+^ cells would attenuate binge-type eating.

## Materials and methods

### Animals and housing

Adult male and female heterozygous PACAP-Cre transgenic (Adcyap1-2A-Cre) mice or wild-type littermate mice on a C57BL/6 J background (*N* = 70, 35 male, 35 female; 23 wild-type, 47 PACAP-Cre) were individually housed starting at 7—11 weeks old, one week before the start of testing, in an AAALAC accredited facility, on a 12-h reversed light/dark cycle, with lights off at 0600 h. PACAP-Cre mice were bred in-house from mice originally purchased from Jackson Laboratory (JAX stock #030155, Bar Harbor, ME, USA) (Harris et al. 2014; Pauli et al. 2022). All mice received ad libitum water and chow (Laboratory Rodent Diet 5001, Lab Diet, St. Louis, MO, USA) throughout the study. Experiments were approved by the Institutional Animal Care and Use Committee of Drexel University College of Medicine and complied with the ARRIVE guidelines, carried out in accordance with the National Institutes of Health Guide for the Care and Use of Laboratory Animals (National Research Council (US) Committee for the Update of the Guide for the Care and Use of Laboratory Animals 2011).

## Detailed methods

**Fig. 1:**
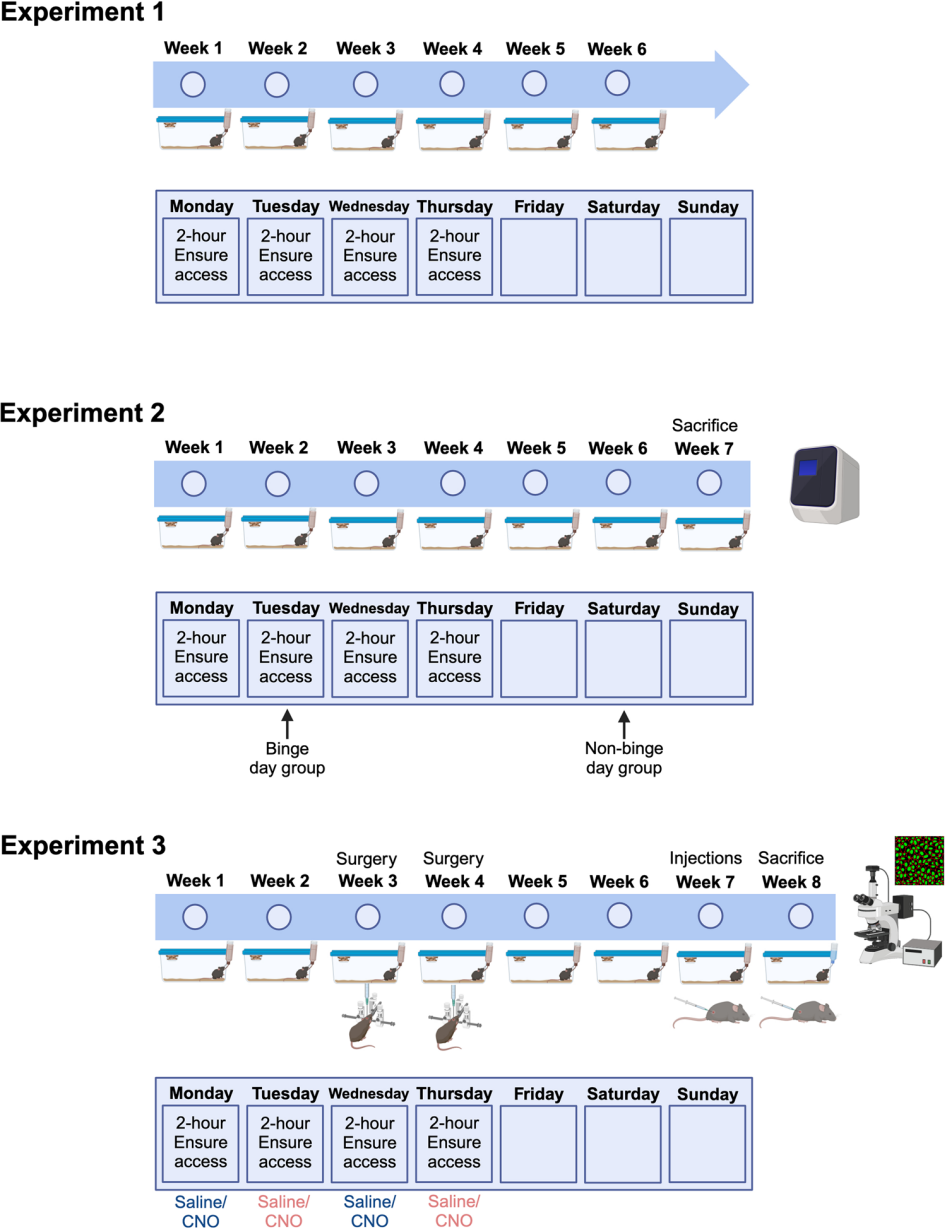
Experimental timelines. In *Experiment 1*, mice were given 2-h access to Ensure (or no access), 4 days per week for 6 weeks, and intake and body weight were measured. In *Experiment 2*, mice from Experiment 1 were sacrificed during the 7th week of the binge-type eating paradigm, immediately prior to the start of the binge session (binge day group or Control) or at the equivalent time of a day without Ensure access (non-binge day group or Control), and their gene expression in the paraventricular nucleus of the thalamus (PVT) was examined using quantitative real-time polymerase chain reaction. In *Experiment 3*, mice underwent the binge-type eating paradigm, were injected in the PVT with a Cre-dependent excitatory DREADD or control virus during the 3rd or 4th week of the paradigm, and then were injected with clozapine n-oxide (CNO) or saline vehicle during the 7th week of the paradigm, 30 min prior to their binge eating session. After 48 h, they were injected with the other drug, 30 min prior to their binge eating session. Intake was recorded after the injections. During the 8th week of the paradigm, a subset of the mice was injected between-subject with CNO or saline and sacrificed after 120 min. Created with BioRender.com

See Fig. 1 for experimental timelines.

### Experiment 1 – Model of binge-type eating

#### Experimental protocol

Male and female mice (of both wild-type and PACAP-Cre genotypes) were given 2-h access to Ensure, 4 days per week for 6 weeks (Binge group; *n* = 12/sex) or were not given access to Ensure (Control group; *n* = 12 females, *n* = 8 males). Ensure, chow, and total kcal intake were analyzed as calories normalized to body weight in grams [(kcal / g BW) * 100]. Preference was calculated by dividing the calories of Ensure consumed by total calories consumed (Ensure + chow) during the binge session and then multiplying by 100, for each animal per binge session [(2-h Ensure kcal / 2-h total kcal) * 100]. Binge score was calculated by subtracting the average 2-h chow intake of the male mice in the Control group or female mice in the Control group from the Ensure intake of each male mouse in the Binge group or female mouse in the Binge group, respectively [(individual subject Ensure intake) – (average control group 2-h chow intake)]. Weight gain was calculated as percent change from body weight in Week 1.

#### Binge-type eating paradigm

Starting 4 h after lights off, ad libitum fed mice were given 2-h access to Milk Chocolate Ensure Plus® (Abbott Nutrition, East Windsor, NJ, USA) in 16-oz bottles with non-drip sipper tubes. This was repeated for 4 days per week (Monday—Thursday). The Ensure contained 1.32 kcal/g and was comprised of 57% carbohydrate (23% sucrose), 28% fat, and 15% protein. The chow diet (Laboratory Rodent Diet 5001) contained 3.36 kcal/g and was comprised of 58% carbohydrate (8% sucrose), 13% fat, and 29% protein. Chow, water, and Ensure, when present, were weighed before and after each session. Body weight was measured at least once each week. A 4500 Hz tone was played immediately before delivery of Ensure to each mouse, to cue the beginning of the binge eating session.

#### Statistical analyses

A mixed ANOVA was used to compare Ensure intake to chow intake (kcal) across 2-h sessions on access days, and to analyze Ensure intake (kcal/body weight), binge score, 2-h chow intake (kcal/body weight), 24-h chow intake (kcal/body weight), total caloric intake (kcal/body weight), and body weight, with group and/or sex as between-subject measure(s) and day or week of measurement as the within-subject measure. Linear regression was used to examine the relationship between Ensure intake and access day. Significant main effects were followed up with a Sidak pairwise comparison test. Sphericity was determined using Mauchly's test, and a Greenhouse–Geisser correction was used when sphericity was violated. Significance was determined at *p* < 0.05. Effect size was determined with partial eta squared. Analyses with non-access day intake lacked data from six females and two males from the Control group and two males and two females from the Binge group. Analyses also lacked data from two females and two males from the Binge group for 24-h chow intake (24-h chow and 24-h kcal).

### Experiment 2 – PACAP gene expression in the PVT relative to binge-type eating

#### Experimental protocol

Male and female mice from the Binge group (*n* = 6/sex/group) in Experiment 1 were sacrificed following the 6th week of the binge-type eating paradigm, immediately prior to the start of the binge session (binge day group), or at the equivalent time on a day without Ensure access (non-binge day group). A subset of the male and female Control mice (*n* = 6/sex) from Experiment 1 was sacrificed at the same time of day and days of the week as the binge and non-binge day groups. These time points were selected to identify changes in PACAP mRNA relative to the start of an Ensure consumption session, and to distinguish this from effects of history of exposure to Ensure. Synthesis of mRNA can be detected in fewer than 30 min following a stimulus (Ben-Ari et al. 2010; Muller et al. 2024; Perez-Ortin 2007). Sacrifice was via rapid decapitation. mRNA was examined using qRT-PCR.

#### Quantitative real time PCR (qRT-PCR)

Immediately after sacrifice, the brain was placed in a matrix slicing guide, with the ventral surface facing up. Two coronal cuts were made, at the middle optic chiasm and 1.0 mm caudal to this, yielding one brain slice (Bregma − 0.75 mm to − 1.75 mm) (Paxinos and Franklin 2004). This slice was then placed under a microscope on a petri dish filled with ice, and the whole PVT was dissected out, as an inverted isosceles triangle below the third ventricle, approximately 0.3 mm wide at the base. Brain sections were stored at − 20 °C in RNA*later* (Qiagen Inc., Valenia, CA, USA) until extraction of RNA. Total RNA from each brain sample was extracted using an RNeasy Mini Kit (Qiagen Inc.), with DNA removed using RNase-free DNase 1 (Qiagen Inc.). The yield was quantified with a NanoDrop Lite spectrophotometer (Thermo Electron North America LLC, Madison Wisconsin), with A260/A280 ratios between 1.85 and 2.27, indicating high purity. cDNA was then reverse transcribed using SuperScript® VILO™ Master Mix (Invitrogen, Grand Island, NY, USA) in a SimpliAmp™ Thermal Cycler (Applied Biosystems, Waltham, MA, USA), using 1 μg of RNA from each sample. For qRT-PCR, a SYBR Green PCR core reagents kit was used (Applied Biosystems, Grand Island, NY, USA), with 12.5 ng of cDNA template in a 25 μl reaction volume in MicroAmp® Fast Optical 96-Well Reaction Plates (Applied Biosystems). A StepOnePlus Real-Time PCR System (Applied Biosystems) was used to carry out the reaction, which was run under the conditions of 2 min at 50 °C (primer annealing), 10 min at 95 °C (polymerase activation and sequence extension), and 40 cycles of 15 s at 95 °C (denaturation) plus 1 min at 60 °C (annealing and extension). Each sample was run in triplicate, and each run included a no-template control. Target gene expression was quantified using the relative quantification method (ΔΔC_T_), with cyclophilin-A as the housekeeping gene. Primer sequences were: cyclophilin-A forward: 5′-ATTCATGTGCCAGGGTGGTG-3′, reverse: 5′-TGCCAGGACCTGTATGCTTT-3′; PACAP forward: 5′-GACCAGAAGACGAGGCTTACG-3′, reverse: 5′-GTCCGCTGGATAGTAAAGGGC-3′. Both primers were used at a 200 nM concentration. The primers were designed with the NCBI Primer design tool (http://www.ncbi.nlm.nih.gov/tools/primer-blast/) (Ye et al. 2012), and purchased from Invitrogen at ThermoFisher Scientific (Grand Island, NY, USA).

#### Statistical analyses

A two-way ANOVA with group and sex as between-subjects measures was used to examine levels of PACAP mRNA in the PVT. A Pearson product-moment correlation was used to determine the relationship between levels of PACAP mRNA (ΔC_T_ value) and average 2-h intake (kcal/body weight). In light of our prior observations of sex differences in PACAP expression in the PVT (Curtis et al. 2023), an a priori decision was made to compare males in the non-binge day group to females in the Control group, using an independent *t*-test. Significant main effects were followed up with a Sidak pairwise comparison test. Sphericity was determined using Mauchly's test. Significance was determined at *p* < 0.05. Effect size was determined with partial eta squared.

### Experiment 3 – Effects of PACAP^+^ cell activity in the PVT on binge-type eating

#### Experimental protocol

Male and female PACAP-Cre transgenic mice (*n* = 15 males, 11 females) underwent the binge-type eating paradigm (see Experiment 1), and received injections into the PVT of a Cre-dependent excitatory DREADD (*n* = 14) or control virus (*n* = 12) during the 3rd or 4th week of the paradigm. Following 6 total weeks of binge-type eating (to match the duration of Ensure exposure in Experiment 2), all mice received intraperitoneal (i.p.) injections of 5 mg/kg clozapine n-oxide (CNO; HelloBio, Princeton, NJ, USA) or saline vehicle (~ 0.14 mL; Baxter International Inc., Deerfield, IL, USA), 30 min prior to their binge eating session, and subsequent intake was recorded. These injections were made within-subject, with 48 h between injections. One week later, to ensure that Cre was co-expressed with PACAP peptide, and that injection of CNO resulted in excitation of these Cre^+^ cells, a subset of the mice was injected between-subject with CNO or saline and sacrificed 120 min later. This timing was selected to allow 30 min for CNO to reach peak levels (MacLaren et al. 2016) and an additional 90 min for c-Fos to reach peak levels (Kovacs 2008). Immunohistochemistry was then used to detect Cre, PACAP, and c-Fos. Injection placement was evaluated in all mice.

#### Stereotaxic surgery

Mice were injected with 0.25 µL of Cre-dependent excitatory DREADD (pAAV8-hSyn-DIO-hM3D(G_q_)-mCherry, Addgene, Watertown, MA, USA) or control virus (pAAV8-hSyn-DIO-mCherry, Addgene) counterbalanced across sexes (male: *n* = 7 – 8/viral injection; female: *n* = 5 – 6/viral injection) into the middle / posterior PVT. Viral titer was a minimum of 4 × 1012 vg/mL. They were anesthetized in an induction chamber with 5% isoflurane in 2 L/min oxygen and then maintained under anesthesia through a nosecone with 1—2% isoflurane in 1 L/min oxygen. Warm saline (1 ml s.c., Baxter International Inc.) was injected to prevent dehydration, and bupivacaine (2 mg/kg s.c., Hospira Worldwide, Lake Forest, IL, USA) was injected into the scalp prior to incision. A 10 µL Nanofil syringe (World Precision Instruments, Sarasota, FL, USA) was lowered into the PVT (AP: -1.6, ML: -0.1, DV: -3.4, at a 6° angle) and the virus was injected at a rate of 50 nL/min. The syringe was then left in place for 10 min to allow for diffusion. Incisions were closed using wounds clips (Brain Tree Scientific Inc., Braintree, MA, USA), and lidocaine ointment and antibiotic ointment were applied to the incisions. Buprenorphine hydrochloride (0.03 mg/kg s.c., Reckitt & Colman Inc., Slough, UK) was administered for post-operative analgesia. Animals were handled and monitored during the week of recovery from surgery.

#### Immunohistochemistry

Mice were deeply anesthetized with Euthasol solution C3N (390 mg/kg pentobarbital sodium/ 50 mg/kg phenytoin sodium; Virbac, Fort Worth, TX, USA) (i.p.) and perfused transcardially with 200 ml of ice-cold 0.9% sodium chloride followed by 200 ml of 4% paraformaldehyde in 0.1 M phosphate buffer, pH 7.4. Brains were then removed, post-fixed in 4% paraformaldehyde for 24 h at 4 ºC, cryoprotected in 30% sucrose for 2 − 4 days at 4 ºC, and then frozen and stored at − 80 ºC. They were sliced coronally on a cryostat at 30 μm, and the sections were stored at − 20 °C in antifreeze solution (37.5% ethylene glycol, 20% sucrose in 0.03 M PBS). Every sixth section through the PVT was taken for processing and analysis, resulting in approximately 8 sections per brain.

To label PACAP, Cre, and c-Fos, free-floating sections were rinsed for 10 min in 0.1% hydrogen peroxide to remove endogenous peroxidase activity. After rinsing in 0.1 M PBS, they were blocked for 90 min in 5% normal donkey serum containing 0.5% Triton X-100 in PBS and incubated overnight at 4 ºC in polyclonal rabbit anti-PACAP-27/38 (1:300, Bioss Antibodies, Woburn, MA, USA; cat# BS0190R, lot# AI11065308), polyclonal guinea-pig anti-Cre (1:500, Synaptic Systems, Goettingen, Germany; cat# 257 004, lot# 3–17), and polyclonal sheep anti-c-Fos (1:1000, Osenses, Keswick, Australia, cat# OSC00007W, lot# YF3934611). After the antibody incubation, the slices were rinsed in PBS and incubated for 2 h in donkey anti-rabbit (Alexa Fluor® 405) (1:200, Abcam, Cambridge, MA, USA; cat# ab175651, lot# GR3444080–1), donkey anti-guinea pig (Alexa Fluor® 647) (1:200, Jackson ImmunoResearch Labs, West Grove, PA, USA, cat# 706–605-148, lot# 140553), and donkey anti-sheep (Alexa Fluor® 488) (1:200, Abcam, Cambridge, MA, USA; cat# ab150177, lot# GR3441849-2). Pilot experiments were run to determine optimal staining conditions, and alternate sections did not show immunofluorescence when processed with the primary or secondary antibody omitted. All sections were processed at the same time under the same conditions. Sections were then mounted on slides and dried overnight in the dark, coverslipped with ProLong® Diamond Antifade Mountant (Life Technologies, Carlsbad, CA, USA), and allowed to set for 24 h before imaging.

Imaging was conducted with a Leica DM5500 automated microscope (Buffalo Grove, IL, USA). The images were captured with an Olympus DP71 high resolution digital color camera (Waltham, MA, USA) with Slidebook V6 image acquisition and analysis software (3i, Denver, CO, USA). Exposure conditions were the same for each slice processed with each primary antibody. Representative images were taken with a Leica DM6B Thunder episcope or a Leica SP8 VIS/405 HyVolution confocal microscope (Buffalo Grove, IL, USA). For analysis, all counting was performed manually, by an evaluator blind to the condition of the subject.

#### Histology

Injection placement was confirmed by slide mounting and coverslipping alternate sections to those used for immunohistochemistry, and imaging them with a Leica DM5500 automated microscope. Images were captured with an Olympus DP71 high-resolution digital color camera (Waltham, MA, USA) with Slidebook V6 image acquisition and analysis software (3i, Denver, CO, USA). Representative images were taken with a Leica SP8 VIS/405 HyVolution confocal microscope.

#### Statistical analyses

An independent *t*-test was used to compare immunohistochemical labeling between males and females, and between animals injected with CNO and those injected with saline. A mixed ANOVA, with virus and sex as the between-subjects measures and drug injection as the within-subjects measure, was used to examine Ensure and chow intake on injection day. Significant main effects in all experiments were followed up with a Sidak pairwise comparison test. Sphericity was determined using Mauchly's test. Significance was determined at *p* < 0.05. Effect size was determined with Cohen’s d or partial eta squared. Three mice were removed from analysis from the Control group and five from the G_q_ group due to off-target injections (more than 0.3 mm lateral to the target region or into the anterior PVT) or no evidence of successful injection.

## Results

### Experiment 1 – Model of binge-type eating

#### Intake during a 2-h period

In the Binge group, there was significantly greater consumption of Ensure than chow during the Ensure access period (*F*_1, 32_ = 531.96, *p* < 0.001, η_p_^2^ = 0.94) (data not shown). Males demonstrated an 87.8% preference and females demonstrated a 90.2% preference for Ensure. Due to this high preference, further analyses of intake in the Binge group during the 2-h Ensure access session examined only Ensure consumption.

**Fig. 2:**
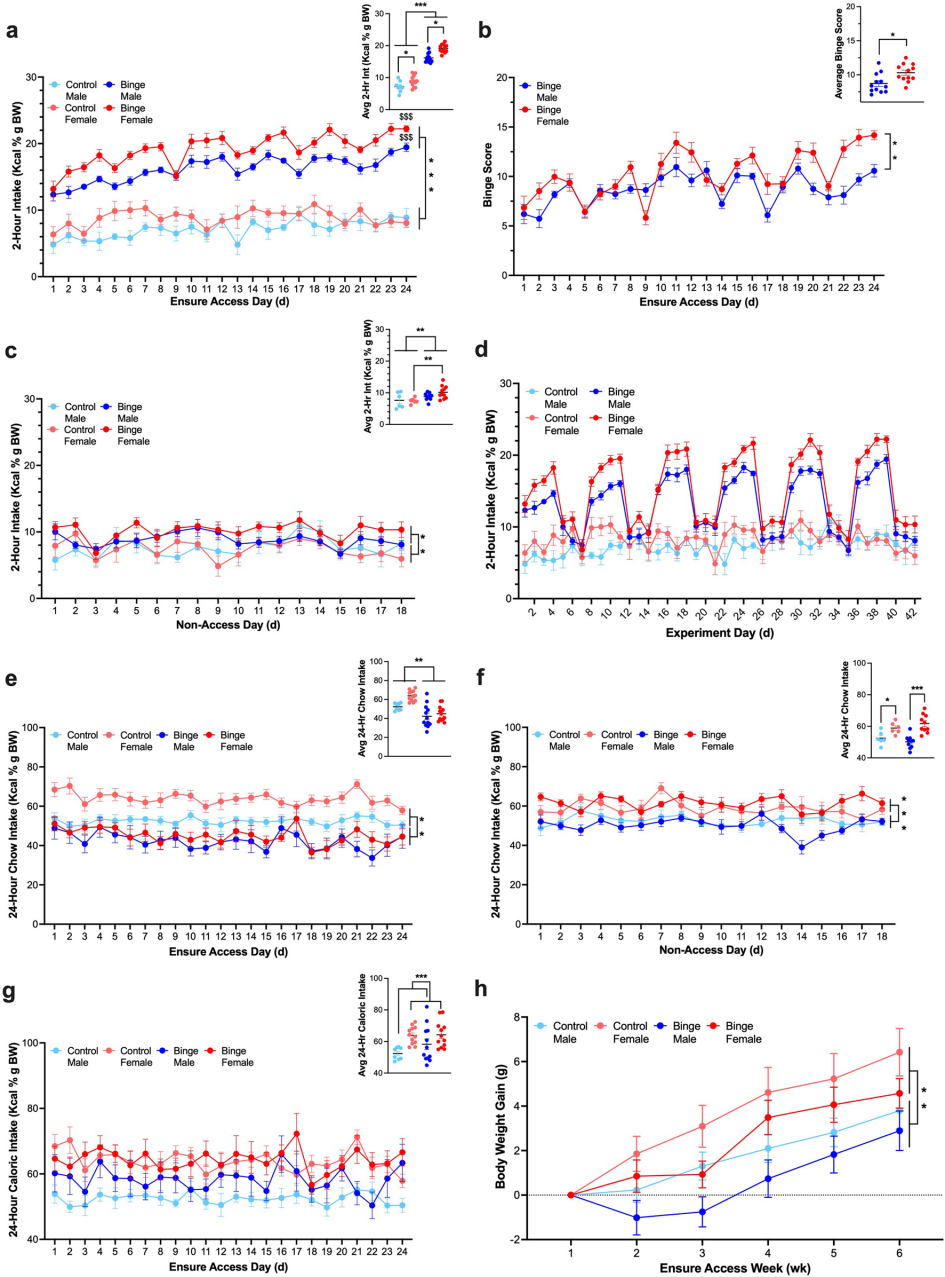
Model of binge-type eating (Experiment 1). **a** For 2-h intake on days with Ensure access, the Binge group consumed more calories per body weight than the Control group, with Binge intake increasing over the course of the experiment, and females in both the Binge and Control groups consuming more than males in the respective groups. Inset is average 2-h intake for each subject across all access days. **b** Binge scores increased over the course of the experiment, and females had higher binge scores than males. Inset is average binge score for each subject across all access days. **c** For 2-h chow intake on days without Ensure access, the Binge group consumed more calories of chow per body weight than the Control group, and females in the Binge group consumed significantly more calories per body weight than females in the Control group. Inset is average 2-h chow intake for each subject across all days Ensure access. **d** For 2-h intake across both days with Ensure access and days without Ensure access, intake varied in the Binge group depending on access. **e** For 24-h chow intake on days with Ensure access, the Binge group consumed fewer calories of chow per body weight than the Control group, and females consumed more than males. Inset is average 24-h chow intake for each subject across all access days. **f** For 24-h chow intake on days without Ensure access, females consumed significantly more calories per body weight than males. Inset is average 24-h chow intake for each subject across all days without Ensure access. **g** For 24-h calories consumed on days of Ensure access, females consumed significantly more total calories per body weight than males. Inset is average 24-h calories consumed for each subject across all days of Ensure access. **h** Body weight increased over the course of the experiment, with females gaining more weight relative to their initial body weight than males. Values are Mean ± S.E.M. ^$$$^*p* < 0.001 vs Day 1, ***p < 0.001, **p < 0.01, *p < 0.05

Examining 2-h intake on days with Ensure access, comparison of Ensure consumption in the Binge group to chow intake at the same time in the Control group revealed a main effect of access day (*F*_11.16, 412.94_ = 11.06, *p* < 0.001, η_p_^2^ = 0.23), group (*F*_1, 37_ = 427.97, *p* < 0.001, η_p_^2^ = 0.92), and sex (*F*_1, 37_ = 31.20, *p* < 0.001, η_p_^2^ = 0.46), as well as an interaction effect between access day and group (*F*_11.16, 412.94_ = 4.03, *p* < 0.001, η_p_^2^ = 0.10) (Fig. 2a). Pairwise comparisons between days showed that both male and female mice in the Binge group increased their intake over the course of the experiment, having significantly higher intake of Ensure calories per body weight on the final day than on the first day (*p* < 0.001). Regression analysis of Ensure intake in males revealed a significant linear model (*F*_1, 23_ = 46.01, *p* < 0.001), with an *R*^*2*^ of 0.68, indicating that access day explained approximately 68% of the variance in Ensure consumption (data not shown). The regression equation was: Ensure consumption = 13.37 + 0.22(access day), confirming that there was an escalation of Ensure intake over time for males. Regression analysis of Ensure intake in females also revealed a significant linear model (*F*_1, 23_ = 31.48, p < 0.001), with an *R*^*2*^ of 0.59, indicating that access day explained 59% of the variance in Ensure consumption (data not shown). The regression equation for females was: Ensure consumption = 15.94 + 0.26(access day), confirming that there was also an escalation of Ensure intake over time for females. In contrast, in the Control group, there was no difference in intake on the first day compared to the final day (*p* = 0.650). Mice in the Binge group consumed significantly more Ensure during the 2-h session compared to mice in the Control group during the same 2-h session (*p* < 0.001), and pairwise comparisons between sexes revealed that females compared to males in the Binge group consumed significantly more Ensure per body weight (*p* < 0.001), and females compared to males in the Control group consumed significantly more chow per body weight (*p* = 0.003) (Fig. 2 (a, inset)). Examining binge scores also revealed a significant main effect of access day (*F*_7.66, 168.51_ = 15.41, *p* < 0.001, η_p_^2^ = 0.41) and sex (*F*_1, 22_ = 8.20, *p* = 0.009, η_p_^2^ = 0.27), as well an interaction effect between access day and sex (*F*_7.66, 168.51_ = 3.64, *p* < 0.001, η_p_^2^ = 0.14). Pairwise comparisons between days showed that binge score increased over the course of the experiment (*p* < 0.001) (Fig. 2b). Females demonstrated significantly greater binge scores than males (*p* = 0.009) (Fig. 2 (b, inset)). These results suggest that on days of access to Ensure, both male and female mice engage in binge-type eating, and that females binge to a greater extent than males.

Examining 2-h intake on days without Ensure access also revealed a main effect of day (*F*_17, 493_ = 2.97, *p* < 0.001, η_p_^2^ = 0.09), but there was no discernable pattern of increase or decrease across time (Fig. 2c, d). There was a main effect of group (*F*_1, 29_ = 9.72, *p* = 0.004, η_p_^2^ = 0.25), with the Binge group consuming significantly more calories of chow per body weight than the Control group (*p* = 0.004). There was no main effect of sex (*F*_1, 29_ = 0.93, *p* = 0.342, η_p_^2^ = 0.03) and no interaction effect between sex and group (*F*_1, 29_ = 1.53, *p* = 0.227, η_p_^2^ = 0.05); however, comparisons of simple main effects revealed that, in females, the Binge group consumed significantly more per body weight during the 2-h session on non-access days than the Control group (*p* = 0.004). This effect was not found in males (*p* = 0.197) (Fig. 2 (d, inset)). These results suggest that, on days without Ensure access, Binge female but not Binge male mice continue to engage in greater 2-h intake than Controls.

#### Intake during a 24-h period

Examining 24-h chow intake on days with Ensure access revealed a main effect of access day (*F*_9.72, 389.15_ = 2.16, *p* = 0.021, η_p_^2^ = 0.05); however, there was again no discernable pattern of intake. There was also a main effect of group (*F*_1, 40_ = 32.62, *p* < 0.001, η_p_^2^ = 0.45) and a main effect of sex (*F*_1, 40_ = 7.88, *p* = 0.008, η_p_^2^ = 0.17), but no significant interaction effect between group and sex (*F*_1, 40_ = 53.00, *p* = 0.091, η_p_^2^ = 0.07) (Fig. 2e). Females consumed more calories from chow per body weight than males (*p* = 0.008), and mice in the Binge group consumed significantly fewer calories from chow than mice in the Control group (*p* < 0.001) (Fig. 2 (e, inset)).

Examining 24-h chow intake on days without Ensure access, there was a main effect of day (*F*_17, 306_ = 1.98, *p* = 0.012, η_p_^2^ = 0.10) and an interaction effect between day and group (*F*_17, 306_ = 1.78, *p* = 0.030, η_p_^2^ = 0.09); however, there was no discernable pattern of increase or decrease over time. There was again a main effect of sex (*F*_1, 18_ = 5.43, *p* < 0.001, η_p_^2^ = 0.64), with females consuming significantly more calories per body weight from chow than males (*p* < 0.001). There was no main effect of group (*F*_1, 18_ = 0.09, *p* = 0.767, η_p_^2^ = 0.01), although there was a significant interaction effect between group and sex (*F*_1, 18_ = 5.43, *p* = 0.032, η_p_^2^ = 0.23) (Fig. 2f). Pairwise comparisons indicated that females in the Binge group consumed more chow than males in the Binge group (*p* < 0.001), and females in the Control group consumed more chow than males in the Control group (*p* = 0.028) (Fig. 2 (f, inset)). These chow results together indicate that mice in the Binge group may have reduced chow intake on days of Ensure access, but they consume the same amount as those in the Control group on days without Ensure access.

Examining 24-h calories consumed (inclusive of both chow and Ensure, when relevant) on days of Ensure access, there was a main effect of sex (*F*_1, 40_ = 12.13, *p* < 0.001, η_p_^2^ = 0.23), with females consuming significantly more total calories per body weight than males (Fig. 2 (g, inset)). There was no main effect of access day (*F*_9.85, 393.82_ = 1.11, *p* = 0.324, η_p_^2^ = 0.03) and no main effect of group (*F*_1, 40_ = 1.57, *p* = 0.218, η_p_^2^ = 0.04) (Fig. 2g). These results suggest that, although mice in the Binge group consume significantly more calories during their binge sessions, this does not result in general overeating.

#### Body weight

Examining change in bodyweight over the course of the experiment, there was a main effect of week (*F*_2.99, 119.92_ = 42.26, *p* < 0.001, η_p_^2^ = 0.51), with weight increasing over the course of the experiment. There was a trend for a main effect of group (*F*_1, 40_ = 3.63, *p* = 0.064, η_p_^2^ = 0.08) and a significant main effect of sex (*F*_1, 40_ = 8.47, *p* = 0.006, η_p_^2^ = 0.18), but there was no interaction effect between group and sex (*F*_1, 40_ = 0.01, *p* = 0.918, η_p_^2^ = 0.00). The animals in the Control group showed a trend for more weight gain than animals in the Binge group (*p* = 0.064), and females gained more weight relative to their initial body weight than males (*p* = 0.006) (Fig. 2h). These results suggest that, despite their greater consumption of calories in the 2-h sessions, the Binge group did not gain significantly more weight than the Control group.

### Experiment 2 – PACAP gene expression in the PVT relative to binge-type eating

**Fig. 3:**
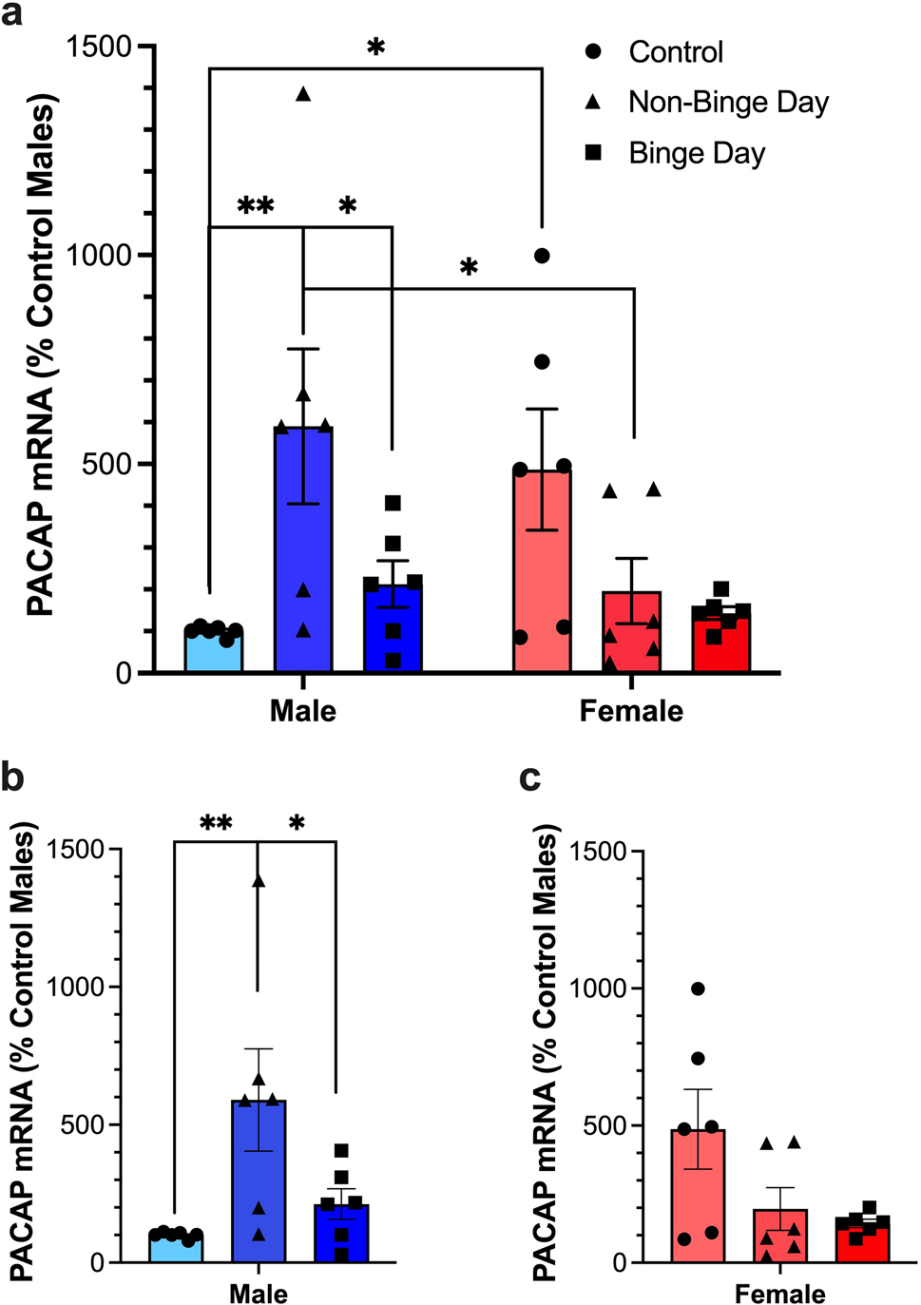
Gene expression of PACAP in the PVT relative to binge-type eating (Experiment 2). **a** Between the Control groups, females had significantly higher levels of PACAP mRNA than males. Non-binge day males had significantly higher levels of PACAP mRNA than non-binge day females. Males in the non-binge day group had levels of PACAP mRNA that were statistically no different than Control females. **b** Among males, the non-binge day group had significantly higher levels of PACAP mRNA than the Control group, but the binge day group had significantly lower levels of PACAP mRNA than the non-binge day group. **c** Among females, there were no significant differences between groups. Values are Mean ± S.E.M. **p < 0.01, *p < 0.05

Examining levels of PACAP mRNA in the PVT, results revealed no main effect of group (*F*_2, 30_ = 2.16, *p* = 0.133, η_p_^2^ = 0.13) or sex (*F*_1, 30_ = 0.09, *p* = 0.762, η_p_^2^ = 0.00), but there was a significant interaction effect between group and sex (*F*_2, 30_ = 7.08, *p* = 0.003, η_p_^2^ = 0.32) (Fig. 3a). Pairwise comparisons revealed that Control females had significantly higher levels of PACAP than Control males (*p* = 0.014), but that non-binge day males had significantly higher levels than non-binge day females (*p* = 0.012), while there was no significant difference between the binge day groups (*p* = 0.641). Levels of PACAP in the non-binge day males were not significantly different than Control females (*t*_10_ = 0.44, *p* = 0.666). For males, pairwise comparisons indicated that the non-binge day group had significantly higher levels of PACAP than the Control group (*p* = 0.007) and that the binge day group had significantly lower levels of PACAP than the non-binge day group (*p* = 0.045) (Fig. 3b). In line with this, while the correlation for all male groups between average 2-h intake and level of PACAP mRNA did not reach significance (*r* = 0.40, *p* = 0.098), the intake-PACAP correlation when including only the Control and non-binge day groups was significant and positive (*r* = 0.64, *p* = 0.025) (data not shown). For females, pairwise comparisons showed a trend for lower levels of PACAP in the binge day group compared to the Control group (*p* = 0.078), but no significant differences between the non-binge day and Control group (*p* = 0.166) or the binge day and non-binge day group (*p* = 0.977) (Fig. 3c). In females, the correlation for all groups between average 2-h intake and level of PACAP mRNA was significant and negative (*r* = -0.60, *p* = 0.008), with a significant negative intake-PACAP correlation being found when including only the Control and binge day groups (*r* = -0.67, *p* = 0.018) (data not shown). These results indicate that, in males but not females, a history of exposure to Ensure under a binge-type eating paradigm elevates levels of PACAP, and that these levels are decreased immediately prior to an Ensure access session.

### Experiment 3 – Effects of PACAP^+^ cell activity in the PVT on binge-type eating

#### Validation of PACAP-Cre mice

**Fig. 4:**
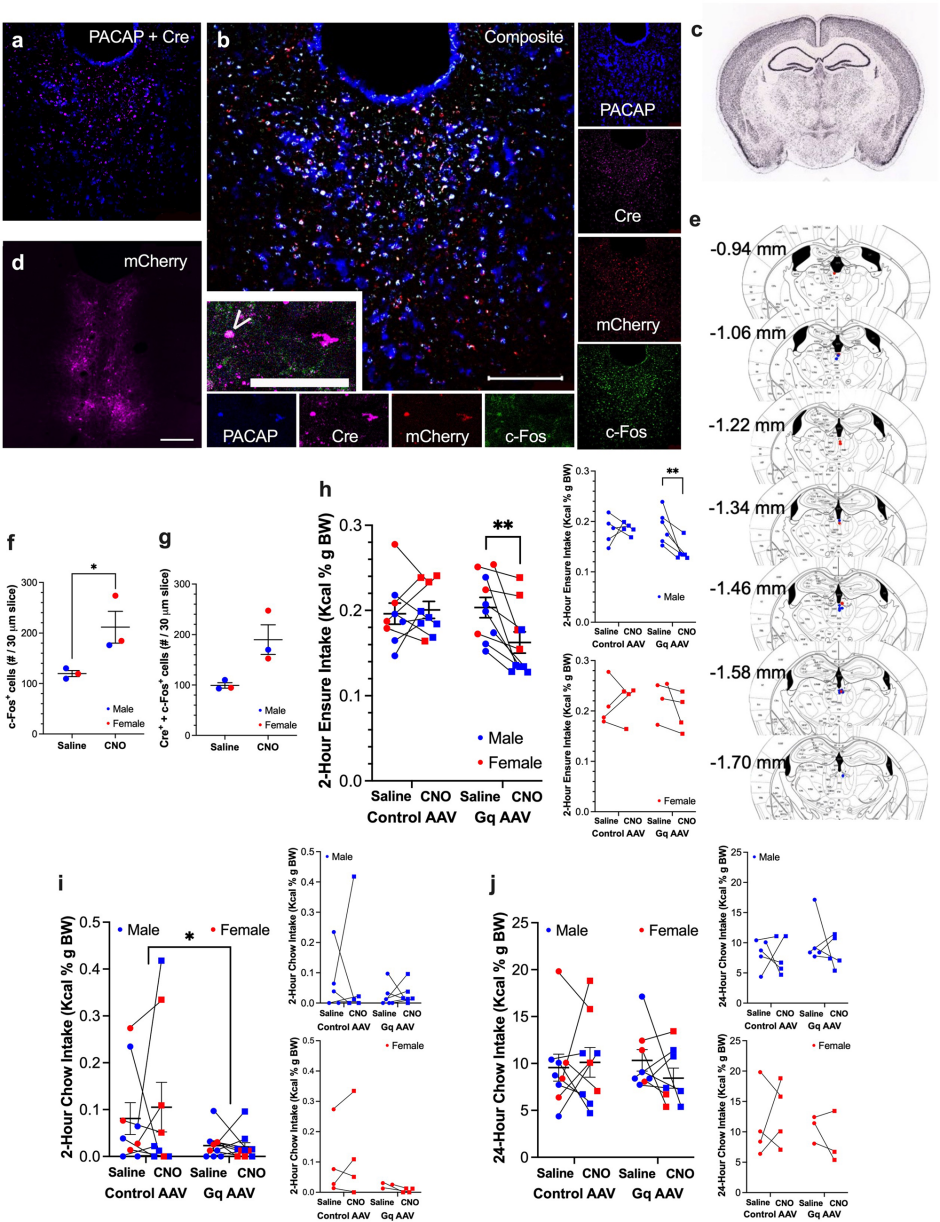
Effects of PACAP^+^ cell activity in the PVT of PACAP-Cre transgenic mice on binge-type eating (Experiment 3). **a** Photomicrograph demonstrating co-labeling of PACAP (blue) and Cre (magenta) in the PVT of PACAP-Cre transgenic mice. **b** Photomicrograph demonstrating co-labeling of PACAP (blue), Cre (magenta), the Cre-dependent excitatory DREADD (pAAV8-hSyn-DIO-hM3D(G_q_)-mCherry) (red), and c-Fos (green) in the PVT of PACAP-Cre transgenic mice after systemic injection with CNO. Inset is higher-magnification image of main image, and shows a cell co-labeled for PACAP, Cre, and AAV (right) and a cell co-labeled for PACAP, Cre, AAV, and c-Fos (left, indicated by “V”). Note that cell size appears to be similar to that in (Gao et al. 2023; Kooiker et al. 2023; Paniccia et al. 2024). Scale bars = 200 μm. **c** Expression of tdTomato in a PACAP-Cre mouse with an Ai14(RCL-tdT) reporter in the Allen Mouse Brain Connectivity Atlas demonstrating detectable levels of expression in the mouse PVT (Allen Institute for Brain Science 2004). **d** Injection track of the excitatory DREADD into the PVT. The AAV here is pseudo-colored magenta. Scale bar = 200 μm. **e** Histological analysis found that virus injections were made between -0.94 and -1.70 mm posterior to bregma, in the middle and posterior subregions of the PVT. Dots indicate placement of on-target injections. Blue = male, red = female. **f** In mice that received the excitatory (G_q_) DREADD, systemic injection with CNO compared to saline resulted in a significant increase in c-Fos in the PVT. **g** In mice that received the excitatory (G_q_) DREADD, systemic injection with CNO compared to saline increased c-Fos labeling in Cre.^+^ neurons in the PVT. **h** For effects of the excitatory (G_q_) DREADD on binge-type eating of Ensure, females overall consumed more Ensure than males, but injection of CNO resulted in reduced consumption of Ensure in the overall G_q_ group, due to a specific decrease in consumption in the males. **i** For effects of the excitatory (G_q_) DREADD on chow intake during the 2-h binge-type eating of Ensure, the overall G_q_ group ate less than the overall control AAV group. **j** For effects of the excitatory (G_q_) DREADD on 24-h chow intake on the day of Ensure access, there were no significant differences between groups. Values are Mean ± S.E.M. **p < 0.01, *p < 0.05

In PACAP-Cre transgenic mice, we observed a high degree of co-localization between PACAP and Cre labeling in the PVT (Fig. 4a). Our analysis showed good penetrance of PACAP^+^ cells (82%), as well as good fidelity (80%). In mice that received the excitatory (G_q_) DREADD in the PVT, systemic injection of CNO compared to saline resulted in a significant increase in c-Fos in the PVT (*t*_4_ = 2.88, *p* = 0.045, *d* = 2.35) (Fig. 4b and f), and 64% compared to 33% of Cre^+^ neurons co-labeled with c-Fos (Fig. 4g). Visual examination of tdTomato gene expression in a PACAP-Cre mouse with an Ai14(RCL-tdT) reporter in the Allen Mouse Brain Connectivity Atlas similarly demonstrated detectable levels of expression in the mouse PVT (Allen Institute for Brain Science 2004) (Fig. 4c). Histological analysis found that virus injections in this experiment were made into the middle and posterior subregions of the PVT, between bregma -0.94 and -1.70 mm (Fig. 4d and e). These results together confirm that PACAP and Cre are co-expressed in the PVT of PACAP-Cre transgenic mice, and that injection of CNO in PACAP-Cre mice that received the excitatory DREADD in the PVT resulted in excitation of PACAP^+^ cells in the PVT.

#### Effects of PACAP^+^ cell activity in the PVT on food intake

Examining effects of the excitatory (G_q_) DREADD on binge-type eating of Ensure, results showed that there was a significant main effect of sex (*F*_1, 15_ = 10.87, *p* = 0.005, η_p_^2^ = 0.42) and a trend for a significant main effect of drug injection (*F*_1, 15_ = 4.35, *p* = 0.054, η_p_^2^ = 0.23), and while there was no significant main effect of virus (*F*_1, 15_ = 1.06, *p* = 0.319, η_p_^2^ = 0.07), there was a significant interaction effect between drug injection and virus (*F*_1, 15_ = 7.03, *p* = 0.018, η_p_^2^ = 0.32) (Fig. 4h). With females overall consuming more Ensure per body weight than males (*p* = 0.005), pairwise comparisons between drug injections in males and females combined revealed that, in the G_q_ group but not in the control AAV group, injection of CNO compared to saline resulted in significantly reduced consumption of Ensure (*p* = 0.004 and *p* = 0.701, respectively). Moreover, tests of simple main effects revealed that this decrease in Ensure intake following CNO in the G_q_ group was due to a significant decrease in Ensure intake in males (*p* = 0.004), but that the change in eating in females did not reach significance (*p* = 0.127) (Fig. 4 (h, right)). In contrast, when examining 2-h chow intake during the same Ensure access sessions, there was no significant main effect of sex (*F*_1, 14_ = 2.39, *p* = 0.144, η_p_^2^ = 0.22) or drug injection (*F*_1, 14_ = 0.56, *p* = 0.468, η_p_^2^ = 0.12), although there was a significant main effect of virus (*F*_1, 14_ = 5.01, *p* = 0.042, η_p_^2^ = 0.26), with the G_q_ group overall eating less chow than the control AAV group overall (Fig. 4i). Examining 24-h chow intake on the injection days, there was no significant main effect of sex (*F*_1, 13_ = 4.04, *p* = 0.066, η_p_^2^ = 0.22), drug injection (*F*_1, 13_ = 0.243, *p* = 0.630, η_p_^2^ = 0.02), or virus (*F*_1, 13_ = 0.030, *p* = 0.865, η_p_^2^ = 0.01) (Fig. 4j). These results suggest that enhancing the activity of PACAP^+^ cells in the PVT decreases eating, particularly binge-type eating of Ensure, and that this specifically occurs in male mice.

## Discussion

The first major result of our study is that we were able to establish our own model of binge-type eating, which could then be used in subsequent experiments. Limited access to Ensure in the home cage (signaled by a cue, 2 h a day, 4 consecutive days per week) induced binge-type eating in male and female mice that met the preclinical criteria for this behavior (Babbs et al. 2012). Specifically, these mice with Ensure access escalated their intake across sessions, with linear regression of intake over access days demonstrating a non-zero slope, as in (Babbs et al. 2012), and they consumed a larger than normal amount of food compared to the Chow control group during the same period of time. Moreover, this occurred to a greater extent in females compared to males. This sex-related difference aligns with previous rodent studies, which found that female compared to male rats were more likely to escalate to extreme progressive ratio self-administration of chocolate flavored sucrose pellets (Spierling et al. 2018) and have higher demand for palatable foods at low cost (Freeman et al. 2021), and that female compared to male C57Bl6/J mice escalated their intake more of palatable food when offered in daily, 2-h sessions (Hildebrandt et al. 2023). It should be noted that the single housing used in our study may itself have facilitated binge-type eating, due to its known impact on stress levels in rodents (Rodriguez-Ortega et al. 2019; Schipper et al. 2018; Solmi et al. 2021). Despite their binge-type eating, our male and female mice did not consume significantly more total calories or gain more weight than our Control mice. This suggests that the binge model did not result in general overeating or excessive weight gain over six weeks of access. Indeed, previous studies of binge-type eating in rodents have noted that weight gain may only occur with sufficient duration of access to palatable food (Berner et al. 2008; Curtis et al. 2019; Kreisler et al. 2017). Thus, with no standard model of binge-type eating in the field, our model of binge-type eating in mice met the preclinical criteria for binge eating and resulted in sex-related differences in behavior that are in line with prior findings.

A second major finding is that PACAP gene expression in the PVT was altered in relation to binge-type eating. Specifically, while females at baseline had higher PACAP levels than males, these levels increased in males, but not females, with a history of exposure to Ensure under a binge-type eating paradigm. This increased PACAP was then reduced immediately prior to an Ensure access session, suggesting that it may endogenously disinhibit binge-type eating in males. Considering the known timing for detection of changes in mRNA (tens of minutes, rather than seconds) (Ben-Ari et al. 2010; Muller et al. 2024; Perez-Ortin 2007), it is possible that females experience a similar decrease in PACAP levels at a later time-point relative to Ensure access sessions. The observed sex-related difference in baseline PACAP gene expression recapitulated our previous findings with PACAP gene expression and peptide levels in the PVT of rats and mice (Curtis et al. 2023). Notably, a history of binge-type eating in males increased PACAP gene expression to levels comparable to those of binge-naïve females. This elevation in PACAP levels in males has previously been observed in another limbic region, the bed nucleus of the stria terminalis, following repeated or chronic exposure to abused drugs or stress (Hammack et al. 2009; Lezak et al. 2014; Miles et al. 2018; Roman et al. 2014). Moreover, previous work has identified a sex-related difference in the role of PACAP in binge-type eating, with injection of PACAP-38 into the ventral tegmental area affecting males but not females (Le et al. 2021). In light of previous work from our lab, which found that temporary inhibition of the PVT decreased PACAP gene expression and increased sucrose seeking in male rats (Gargiulo et al. 2022), we propose that, while female mice are initially more prone to binge eating behavior, perhaps in some way related to their elevated levels of PACAP, males can develop a similar proneness to binge eating as their baseline levels of PACAP increase following chronic or repeated exposure to limited-access palatable food.

A third major finding is that enhanced activity in middle / posterior PVT PACAP^+^ cells prior to the binge session attenuated binge-type eating, and this occurred in males more than in females. This reinforces the idea that a reduction in endogenous levels of PACAP in the PVT could serve to disinhibit binge-type eating. In rats, temporary inhibition of the PVT with the GABA-A agonist, muscimol, has previously been found to promote chow intake (Stratford and Wirtshafter 2013), whereas photoactivation of the anterior PVT abolished sucrose seeking during unexpected reward omission (Do-Monte et al. 2017). While these published findings are consistent with the results of our current study, they contrast with previous findings from our own group, which indicated that intermittent access sucrose drinking was instead enhanced by neuropeptide-specific excitation of the posterior PVT in rats (Barson et al. 2015, 2017). In the present study, of the three mice that were removed from analysis because the excitatory DREADD was injected in the anterior PVT, one responded to CNO injection with a decrease in Ensure intake, one responded with no change in intake, and one responded with an increase in intake. These discrepancies, between studies and between different PVT subregions, may be the result of differences in the access paradigm, type of palatable food, or the specific population of PVT cells being manipulated. It has been hypothesized that disparate results from manipulations of the PVT may be due to variations in cell types and projection targets across the antero-posterior axis of the PVT (Li et al. 2021; McGinty and Otis 2020). A major caveat of the present findings is that PACAP in the PVT is co-expressed with vGLUT2 (Gupta et al. 2018), so chemogenetic excitation of the PACAP^+^ cells likely resulted in the co-release of glutamate, and possibly also other neurochemicals. Thus, we cannot specifically attribute the current behavioral changes to changes in PACAP release. Moreover, with the PVT known to send projections not only to areas of the extended amygdala, such as the central amygdala, bed nucleus of the stria terminalis, and nucleus accumbens, but also within the PVT itself (Li et al. 2021; Vertes and Hoover 2008), it remains to be determined where PACAP from the PVT may act to effect its behavioral changes. Taken together, however, the present data with excitatory DREADDs suggest that the population of PACAP^+^ cells in the PVT could exert an influence on binge-type eating, particularly in male mice. We speculate that, with PACAP expression being higher in the PVT of females, their binge-type eating might be affected by chemogenetic inhibition of this PACAP^+^ cell population.

Overall, the findings from the present experiments indicate that PVT PACAP^+^ cells influence and are influenced by binge-type eating. They suggest that changes in PVT PACAP expression related to binge eating are sex-dependent and occur following a history of exposure to palatable food as well as acutely prior to its intake. They also indicate that PVT PACAP^+^ cell activity inhibits binge-type eating in male mice. Future studies are needed to confirm that this effect is specifically due to the release of PACAP, to identify the specific pathways through which this effect occurs, and to determine if inhibition of PACAP^+^ cell activity can promote binge-type eating. All together, the results suggest that PACAP in the PVT might mediate some of the known involvement of the PVT with palatable food intake.
